# Symptom prevalence, severity, distress and management among patients with chronic diseases

**DOI:** 10.1186/s12912-023-01296-8

**Published:** 2023-05-06

**Authors:** Fatima ALHosni, Mohammad Al Qadire, Omar Al Omari, Huda Al Raqaishi, Atika Khalaf

**Affiliations:** 1 College of Nursing, Sultan Qaboos University, P.O. Box 66, Muscat, PC 123 Sultanate of Oman; 2 Faculty of Nursing, Al Al-Bayt University, P.O. Box 130040, Mafraq, 25113 Jordan; 3 Sultan Qaboos University Hospital, Muscat, Sultanate of Oman; 4 Faculty of Health Sciences, Kristianstad University, Kristianstad, SE-291 88 Sweden

**Keywords:** Symptom prevalence, Severity, Distress, Management, Nonmalignant chronic diseases, Oman

## Abstract

**Background:**

Advanced knowledge, technology, and treatment approaches resulted in longer survival rates for patients suffering from chronic diseases. However, symptoms of these diseases persist and affect the individual’s entire life and normal functioning.

**Aim:**

To assess symptoms prevalence, severity, distress, and management among patients with chronic obstructive pulmonary diseases (COPD), chronic heart failure (CHF), and end-stage renal disease (ESRD) in Oman.

**Design:**

A descriptive cross-sectional design was used.

**Sample and settings:**

The study sample comprised 340 participants who were recruited between May and December 2021 from two referral hospitals and one large dialysis unit in the Sultanate of Oman, Muscat Governate using a convenience sampling technique.

**Results:**

The highly prevalent symptoms among patients with selected chronic diseases were lack of energy (60.9%), pain (57.4%), numbness (53.2%), difficulty sleeping (49.4%), and shortness of breath (45.9%). The most severe symptoms were shortness of breath (53.2%), problems with urination (51.9%), constipation (50.8%), difficulty sleeping (49.7%), and pain (46.2%). The symptom “problems with sexual interests or activity” was found to be the most frequently occurring and highly distressing symptom out of all reported symptoms.

**Conclusions:**

The current study’s findings showed that symptoms were prevalent and that some symptoms were frequent, severe, and highly distressing. In addition, patients perceived symptom treatment as inadequate. Psychological symptoms received less treatment attention compared with physical symptoms. One of the mainstays for managing symptoms can be the introduction of palliative care. Providing palliative care to these patients can alleviate their suffering and improve their quality of life. In addition, designing chronic disease self-management programmes can make a difference in patients’ life.

## Background

In the last decades, the number of people living with chronic diseases has increased dramatically [1]. According to the Centre for Disease Control and Prevention (CDC), 6 out of 10 adults in the United States (US) have chronic diseases, such as heart disease, cancer, chronic lung disease, stroke, Alzheimer’s disease, diabetes, and chronic kidney disease [2]. In Oman, non-communicable diseases are responsible for 72% of total deaths, of which cardiovascular diseases are responsible for 32%, cancer 11%, diabetes 8% and chronic respiratory diseases 2% [3].

Advanced knowledge, technology, and treatment approaches resulted in longer survival rates for patients suffering from acute and chronic diseases [4]. However, the symptoms of these diseases persist and affect the individual’s entire life and normal functioning [4]. These impacts vary from patient to patient based on the disease nature and patient age, and many other factors, such as gender, socioeconomic status, educational level and cultural background [5]. There are numerous chronic diseases that affect humans in different age groups; however, there are certain diseases that are common in adults and are known for their burden [6, 7]. These chronic diseases include, but are not limited to, chronic obstructive lung diseases (COPD), chronic heart failure (CHF) and end-stage renal disease (ESRD). Symptoms such as fatigue, pain, muscle weakness, difficulty breathing, lack of energy, and low mood vary in frequency, severity, and distress among patients with different chronic diseases [6]. Symptoms are dynamic; they change and develop over time as the disease progresses [8]. Furthermore, the patients reported not only physical symptoms but also psychosocial symptoms that affect their independence and psychiatric and spiritual well-being [9]. Therefore, the need to identify the burden of symptoms in patients with chronic obstructive pulmonary diseases (COPD), chronic heart failure (CHF) and end-stage renal disease (ESRD) is very critical and has many implications for healthcare care provision [8].

Multiple studies have been conducted to assess the prevalence of symptoms among patients with chronic obstructive pulmonary disease (COPD), chronic heart failure (CHF), and end-stage renal disease (ESRD) [9–12]. For example, a study was conducted to assess the burden of symptoms among COPD patients and found that patients experienced on average 13 to 15 symptoms [9]. This study reported that the most annoying symptoms were shortness of breath, lack of power, difficulty sleeping, worrying, dry mouth, nervousness and irritability [9]. Another study conducted in Saudi Arabia to assess symptoms among ESRD patients found that the most commonly reported symptoms among all stages of the disease were fatigue (77%), bone pain (60.3%), itching (59.6%) and loss of appetite (50.5%) [10]. Symptom prevalence and distress among patients with nonmalignant chronic diseases have not been adequately explored despite the increasing number of people living with chronic diseases around the world [5]. Experiencing these symptoms increases patient visits to healthcare settings and can cause patient suffering and loss of hope [5]. Additionally, the burden of symptoms can limit individual productivity and their role in the family, which in turn can contribute to additional financial cost [13].

Like other developing countries in the Middle East, there is expected to be an increase in the number of patients with chronic diseases in Oman, particularly chronic obstructive pulmonary diseases (COPD), chronic heart failure (CHF), and end-stage renal disease (ESRD) [3]. This group of people needs quality care and special attention, as they usually live a long time with at least one chronic disease [14]. Regardless of the stage or type of chronic disease, patients experience symptoms due to the disease or its treatment. Assessment of the prevalence, severity and distress of symptoms is essential to understand the burden of symptoms and also to guide healthcare providers and planners [6].

Based on the literature review, different aspects of the knowledge gap that need to be addressed were identified. First, the dimensions of symptoms were not fully explored in most previous studies, as they focused on the prevalence of symptoms and ignored other dimensions, such as severity, frequency, and distress. Second, there is very limited information on the level of symptom treatment as perceived by patients with chronic diseases. Third, no studies have explored the prevalence, severity and distress of symptoms among patients with chronic diseases in Oman. Thus, this study aims to assess symptoms prevalence, severity, distress, and management among patients with chronic obstructive pulmonary diseases (COPD), chronic heart failure (CHF) and end-stage renal disease (ESRD) in Oman.

## Methods

### Design

A descriptive cross-sectional design was used.

### Sample

The study sample comprised 340 participants who were recruited using a convenience sampling technique. We included adult Omani patients who were 18 years or older; diagnosed for more than six months with one of the following diseases: COPD, CHF, or ESRD; currently treated or followed up in one of the selected settings, able to understand Arabic; and agreed to participate in the study.

Those three diseases were selected because they are prevalent, known for their symptom burden that compromise patients’ daily activities, quality of sleep and quality of life [6, 7]. Each disease has symptoms that are influenced by its pathology, and the experience of symptoms associated with them increases patient visits to healthcare settings and can cause patient suffering and loss of hope [5].

However, participants with malignant diseases, fatigued (as stated by the patient), admitted to hospital wards and units, female pregnant patients as pregnancy may affect symptom assessment, patients with a history of cognitive impairment that interferes with their ability to give informed consent, patients with a major change in treatment plan in the previous two weeks (the prescription of new treatment regimen can cause adverse effects/surgical intervention) and patients with highly infectious communicable diseases such as Covid-19 and HIV/AIDS (as it can exaggerate symptoms of chronic disease) were excluded. Furthermore, patients who had more than one of the selected diseases were also excluded to enable the use of the symptom correlated with the selected disease. However, the presence of other chronic disease such as diabetics mellites, ischemic heart disease, stroke, hypertension etc. was documents then categorised as following: patients no additional chronic disease, patient with one, and patients with two or more chronic disease other than CHF, COPD and ESRD.

### Sample size calculation

The sample size calculation was based on the type of analysis that was intended to be conducted. This article presents only part of the original study. The multiple linear regression was planned to determine the variables that predict the level of quality of life (published elsewhere). Based on data from previous studies, the aims of the study, and the data intended to be collected, we expected to include 20 independent variables (m) at maximum. Therefore, the sample size (N) should satisfy both of the following:

1. N 50 + 8 m to test the overall significance of the model (i.e., of R2).
2. N ≥ 104 + m to test the significance of individual independent variables (m = number of independent variables) [15].

Hence, the sample size should be both 50 + 8 * 20 = 210 and 104 + 20 = 124. This sample size allows medium-sized relationships between the independent variables and the dependent or outcome variable, 5% significance, and 80% power, However, to increase the power and generalisability of the results of the study, the number was increased to 340, 120 for ESRD, 120 for CHF, and 100 for COPD.

### Settings

The study was carried out in two hospitals (A (n = 85), and B (n = 135)) and one dialysis centre C (n = 120) in the Sultanate of Oman, Muscat Governorate. The first is a large university affiliated referral hospital with a capacity of 600 beds. The second hospital is a governmental referral hospital. It is a large tertiary-level acute-care hospital operated by the ministry of health of Oman and the bed capacity is 738 [3]. The third is a dialysis centre, which is one of the major dialysis centres in Muscat city. The bed capacity of this centre is 52 and serves 350 patients. Participants were recruited from the outpatient clinics – respiratory clinic (pulmonology), cardiology clinic and nephrology clinic – within these hospitals.

### Instruments

Data were collected using the following instruments:

#### Demographic data sheet (DDS)

The DDS was used to collect demographic data from participants, including age, sex, marital status, educational level, monthly income, work status, and living place. Data on participant health status including diagnosis, comorbidities, number of hospital emergency visits, number of admissions, length of hospital admissions and time since diagnosis were collected from patient medical records.

#### Memorial symptoms assessment scale (MSAS)

The MSAS was developed by Portenoy et al., [16]. It was developed to assess the common physical and psychological symptoms experienced by cancer patients, however, many recent studies have used the scale for the evaluation of symptoms in other chronic diseases, such as COPD [9, 14] and heart failure [17, 18]. This scale is designed to assess symptom prevalence and it assesses symptom severity, frequency, and distress. A Likert scale is used to evaluate each dimension [16]. Each symptom score is an average of its dimensions, and a higher score reflects higher severity, frequency, and distress. The total score in MSAS is the average of the symptom scores for all 32 symptoms [19]. The MSAS includes 32 symptoms in total presented in the form of a questionnaire. The first section consists of 24 symptoms evaluated for severity, frequency, and distress. The second section consists of eight symptoms evaluated only for severity and distress. In addition, participants were asked to indicate if they received treatment for their symptoms and whether this treatment was effective or not.

The Arabic language includes only 30 symptoms; two symptoms were removed during translation: feeling irritable and feeling drowsy. These symptoms were removed as they are synonyms of two other symptoms in the Arabic language: feeling nervous and dizziness [19]. The validity and reliability of the Arabic version were evaluated and established by Abu-Saad Huijer et al., [19] among Lebanese oncology patients. The Cronbach’s α coefficients for the Arabic version of MSAS and its subscales ranged from 0.71 to 0.83. In this study, the Cronbach’s α coefficients were 0.61 for the total score and ranged from 0.74 to 0.81for the subscales.

### Data collection procedure

After obtaining the ethical approval from the selected settings, data collection started. All forms of the instruments, participants’ information sheets, and consent forms were printed. Nurses in charge were met in advance to explain the main purpose of the study and the data collection procedure and obtain their permission. For each data collection visit, patients’ electronic records were checked to confirm the diagnosis prior to patient arrival, and then the patient was met by the researcher to explain the study purpose, procedure, and requirements. A participant information sheet was provided and explained in detail for the participants with each questionnaire. Enough time was given to patients to decide on participation. Once they agreed to participate in the study, a consent form was provided to the participants to be read and signed. Finally, all participants were asked to put the completed questionnaire in a box designated for the purpose within the waiting area. The researcher was available in the area to provide any help for participants if needed.

### Ethical considerations

Ethical approval to conduct the study sought from the ethics committees within the selected settings. The researcher explained the study to the participants, its main purpose, impact and significance, the information required and the time needed to complete the survey. All participants were assured that their participation is voluntary, and they have the right to withdraw from the study at any time and not to answer any research question without affecting their medical and nursing care. Furthermore, a written consent form was obtained from participants. Further, they were informed that no identifiable data is needed, and only aggregated data would be presented or published. Finally, the data was kept in a locked cabinet and on a password-protected computer, and no one outside the research team was allowed to access the data.

### Data analysis

Data were analysed statistically using IBM SPSS software version 23. This software was used for data entry, data cleaning, and data analysis. First, all data for every participant were entered in the software and matched with the data on paper to check for accuracy. Variables with more than 20% missing data were excluded from the final data analysis [20]. This study used descriptive statistics such as frequency, percentage, mean and standard deviation to describe the characteristics of the study sample and to summarise data on the prevalence, frequency, severity, distress and treatment of symptoms.

## Results

### Sample characteristics

#### Demographical characteristics

**Fig. 1:**
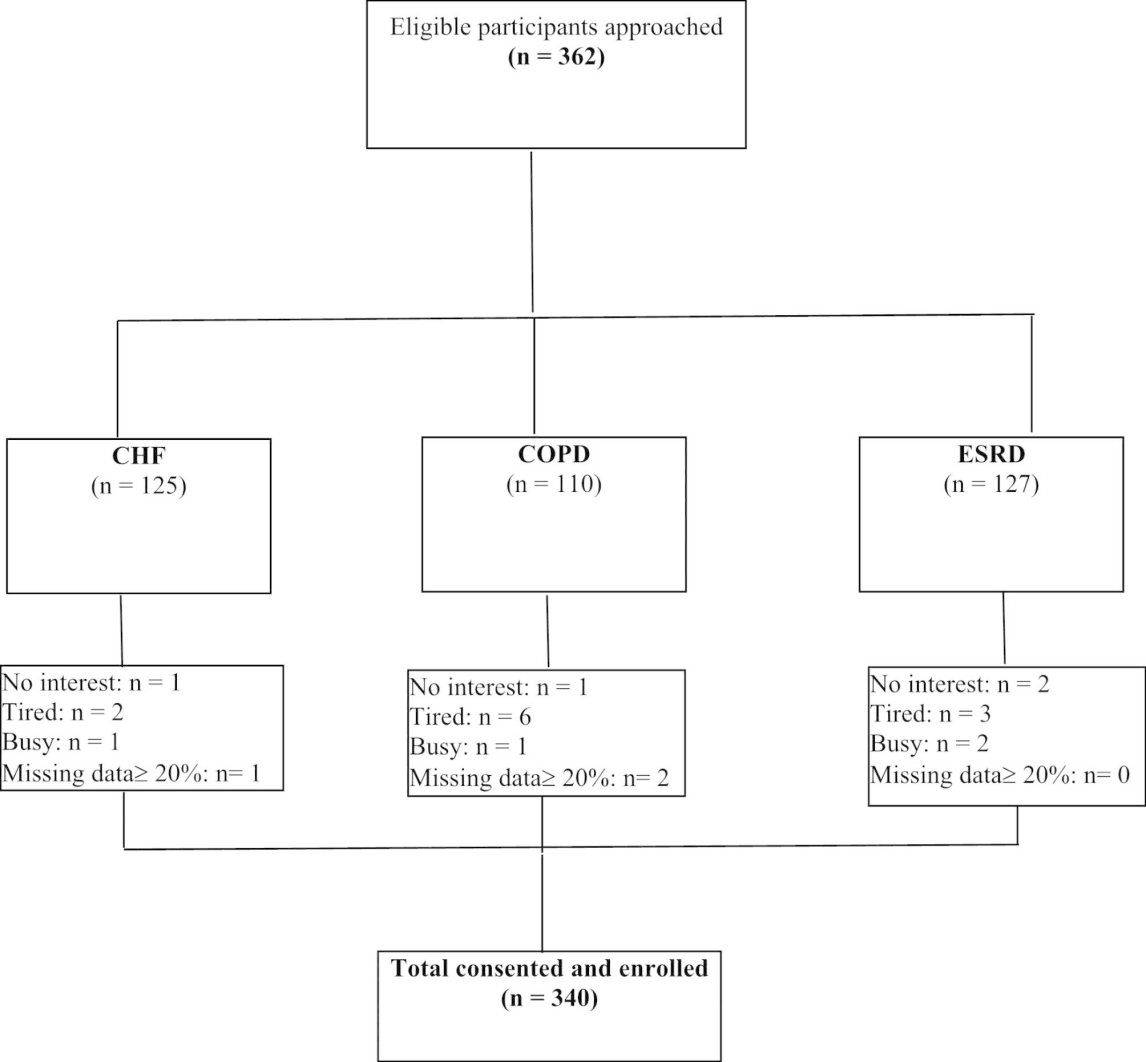
Participants flow diagram. CHF = chronic heart failure, COPD = chronic obstructive pulmonary disease, ESRD = end-stage renal disease

Initially, 362 patients were approached who met the study inclusion criteria. Nineteen patients refused to participate in the study for different reasons, which were lack of interest (n = 8), feeling tired (n = 6), and poor timing or being busy (n = 5). Additionally, three questionnaires had more than 20% missed data and therefore were excluded. There were 340 study participants who completed the survey with a response rate of 94.7% (Fig. 1). They were recruited from three different settings: A (n = 85), B (n = 135) and dialysis unit C (n = 120).

Of the participants, 100 were diagnosed with COPD (29.4%), 120 with ESRD (35.3%) and 120 with CHF (35.3%). The mean age of the participants was 60.6 (*SD* = 14.4) years. Most of the participants were male (63.5%, n = 216). More than two-thirds of the participants were married (71.8%, n = 244). Table 1 shows the demographic characteristics of the participants.

**Table 1:** Participants’ Demographical and Clinical Characteristics (N = 340)

Characteristics	Mean (*SD*)	Min-Max
**Age (years)**	60.6 (14.4)	20–96
**Diagnosis duration (years)**	5.9 (4.6)	0.2–24
**Number of emergency room visits**	1.2 (2.7)	0.0–30
**Number of hospital admissions**	0.4 (0.8)	0.0–9
**Length of Stay (LOS)**	2.5 (9.4)	0.0-120
**The Karnofsky Performance Status Scale (KPSS)**	74 (13.5)	
	**Frequency (%)**	
**Gender**		
Male	216 (63.5)	
Female	124 (36.5)	
**Marital status**		
Married	244 (71.8)	
Not Married	96 (28.2)	
**Educational level**		
Low	299 (87.9)	
High	41 (12.1)	
**Work**		
Working	45 (13.2)	
Not working	295 (86.8)	
**Monthly income**		
1000 OMR or less	281 (82.6)	
More than 1000 OMR	59 (17.4)	
**Medical diagnosis**		
COPD	100 (29.4)	
ESRD	120 (35.3)	
CHF	120 (35.3)	
**Hospital**		
Governmental hospital A	85 (25)	
Governmental hospital B	135 (39.7)	
Dialysis centre C	120 (35.3)	
**Family caregiver**		
Yes	326 (95.9)	
No	14 (4.1)	
**Habits**		
Smoker	58 (17.1)	
Ex-smoker	32 (9.4)	
Regular exerciser	10 (2.9)	
Nonactive	19 (5.6)	
**Chronic diseases**		
No evidence of chronic diseases	62 (18.2)	
One chronic disease	141 (41.5)	
Two or more	137 (40.3)	

#### Clinical characteristics

On average, participants had had their disease for 5.9 (*SD =* 4.6) years. Table 1 illustrates the clinical characteristics of the study participants. Regarding having additional chronic diseases, 141 of the participants reported having one disease (41.5%) and 137 of them reported two or more diseases (40.3%). Only 62 reported having no other chronic diseases (18.2%). Participants reported that disease symptoms resulted in 1.2 (*SD =* 2.7) emergency room visits on average in the last six months. The mean number of hospital admissions due to disease symptoms was reported as 0.4 (*SD =* 0.8) times with an average length of stay 2.5 days (*SD =* 9.4).

### Symptom prevalence

Overall, the prevalence of symptoms among participants in the study sample ranged from 2.4 to 60.9%. The five main symptoms that were prevalent were lack of energy (60.9%), pain (57.4%), numbness (53.2%), difficulty sleeping (49.4%) and shortness of breath (45.9%). Furthermore, 37.6% and 35.3% of the participants reported cough and constipation, respectively. However, the least prevalent symptoms were mouth sores (2.4%), sweats (3.5%), diarrhoea (4.1%), wight loss (4.1%) and difficulty swallowing (4.7%). Table 2 presents pooled symptom prevalence.

**Table 2:** Symptom Prevalence (pooled and by disease)

Symptom	COPD (n = 100)	ESRD (n = 120)	CHF (n = 120)	Total (N = 340)
	Frequency (%)	Frequency (%)	Frequency (%)	Frequency (%)
1. Difficulty concentrating	28 (28)	20 (16.7)	27 (22.5)	75 (22.1)
2. Feeling nervous	17 (17)	35 (29.2)	45 (37.5)	97 (28.5)
3. Feeling sad	16 (16)	40 (33.3)	30 (25)	86 (25.3)
4. Worrying	25 (25)	32 (26.7)	46 (38.3)	103 (30.3)
5. “I don’t look like myself”	12 (12)	36 (30)	25 (20.8)	73 (21.5)
6. Lack of energy	67 (67)	63 (52.5)	77 (64.2)	207 (60.9)
7. Numbness/tingling in hands/feet	52 (52)	61 (50.8)	68 (56.7)	181 (53.2)
8. Dizziness	23 (23)	40 (33.3)	37 (30.8)	100 (29.4)
9. Pain	64 (64)	64 (53.3)	67 (55.8)	195 (57.4)
10. Difficulty sleeping	61 (61)	52 (43.3)	55 (45.8)	168 (49.4)
11. Dry mouth	21 (21)	35 (29.2)	34 (28.3)	90 (26.5)
12. Nausea	8 (8)	40 (33.3)	12 (10)	60 (17.6)
13. Vomiting	1 (1)	24 (20)	2 (1.7)	27 (7.9)
14. Change in the way food tastes	0	14 (11.7)	4 (3.3)	18 (5.3)
15. Lack of appetite	39 (39)	37 (30.8)	22 (18.3)	98 (28.8)
16. Weight loss	1 (1)	9 (7.5)	4 (3.3)	14 (4.1)
17. Difficulty swallowing	2 (2)	9 (7.5)	5 (4.2)	16 (4.7)
18. Cough	62 (62)	28 (23.3)	38 (32.7)	128 (37.6)
19. Shortness of breath	92 (92)	19 (15.8)	45 (37.5)	156 (45.9)
20. Swelling of arms/legs	19 (19)	13 (10.8)	50 (41.7)	82 (24.1)
21. Sweats	0	6 (5)	6 (5)	12 (3.5)
22. Itching	11 (11)	56 (46.7)	19 (15.8)	86 (25.3)
23. Mouth sores	1 (1)	3 (2.5)	4 (3.3)	8 (2.4)
24. Hair loss	0	32 (26.7)	12 (10)	44 (12.9)
25. Changes in skin	1 (1)	25 (20.8)	12 (10)	38 (11.2)
26. Feeling bloated	21 (21)	50 (41.7)	42 (35.0)	113 (33.2)
27. Problems with urination	24 (24)	27 (22.5)	28 (23.3)	79 (23.2)
28. Diarrhoea	3 (3)	8 (6.7)	3 (2.5)	14 (4.1)
29. Constipation	37 (37)	47 (39.2)	36 (30)	120 (35.3)
30. Problems with sexual interest or activity	4 (4)	15 (12.5)	4 (3.3)	23 (6.8)

COPD= chronic obstructive pulmonary disease, ESRD= end-stage renal disease, CHF= chronic heart failure

For COPD patients, the most prevalent symptoms were shortness of breath (92%, n = 92), lack of energy (67%, n = 67), pain (64%, n = 64), cough (62%, n = 62) and difficulty sleeping (61%, n = 61). The least common symptoms were vomiting (1%, n = 1), weight loss (1%, n = 1), mouth sores (1%, n = 1), changes in skin (1%, n = 1) and difficulty swallowing (2%, n = 2). However, there were symptoms that were not reported by any of the participants including change in the way food tastes, sweats and hair loss. Table 2 illustrates symptom prevalence for the three selected chronic diseases (COPD, ESRD and CHF). However, in ESRD participants the most common symptoms were pain (53.3%, n = 64), followed by lack of energy (52.5%, n = 63), numbness (50.8%, n = 61), itching (46.7%, n = 56), and difficulty sleeping (43.3%, n = 52). Moreover, the least prevalent symptoms included mouth sores (2.5%, n = 3), sweats (5%, n = 6), diarrhoea (6.7%, n = 8), difficulty swallowing (7.5%, n = 9) and weight loss (7.5%, n = 9). Finally, the most common symptoms reported by CHF patients (n = 120) were lack of energy (64.2%, n = 77), numbness (56.7%, n = 68), pain (55.8%, n = 67), difficulty sleeping (45.8%, n = 55) and swelling of the arms / legs (41.7%, n = 50). While the least prevalent symptoms among them were vomiting (1.7%, n = 2), diarrhoea (2.5%, n = 3), change in the way food taste (3.3%, n = 4), weight loss (3.3%, n = 4), mouth sores (3.3%, n = 4) and problems with sexual interests (3.3%, n = 4).

### Symptom frequency

In participants who reported having a symptom in MSAS, its frequency was assessed asking participants to rate the frequency of the symptom as rarely, occasionally, frequently, or almost constantly. The frequency of symptoms is presented in Table 3. The most frequently occurring symptom (i.e., constantly) was problems with sexual interest, which was reported by 43.5% of the participants (n = 10). Furthermore, the five main symptoms that were reported to occur frequently included shortness of breath (46.8%, n = 73), feeling bloated (44.2%, n = 50), pain (42.6%, n = 83), lack of appetite (37.8%, n = 37) and difficulty sleeping (35.9%, n = 60). Moreover, the top five symptoms that were reported to occur occasionally included diarrhoea (71.4%, n = 10), vomiting (66.7%, n = 18), sweats (58.3%, n = 7) and both feeling sad and nausea (56.7%, n = 42; 56.7%, n = 34, respectively).

**Table 3:** Pooled symptom frequency based on the Memorial Symptoms Assessment Scale, given in n (%)

Symptom	N	Mode	Rarely	Occasionally	Frequently	Almost constantly
Difficulty concentrating	75	2	20 (26.7)	40 (53.3)	11 (14.7)	4 (5.3)
Feeling nervous	97	2	16 (16.5)	55 (56.7)	16 (16.5)	10 (10.3)
Feeling sad	86	2	12 (14)	42 (48.8)	21 (24.4)	11 (12.8)
Worrying	103	2	15 (14.6)	58 (56.3)	22 (21.4)	8 (7.8)
“I don’t look like myself”	73	-	-	-	-	-
Lack of energy	207	2	11 (5.3)	95 (45.9)	62 (30)	39 (18.8)
Numbness/tingling in hands/feet	181	2	6 (3.3)	94 (51.9)	57 (31.5)	24 (13.3)
Dizziness	99	2	37 (37.4)	40 (40.4)	20 (20.2)	2 (2)
Pain	195	3	12 (6.2)	74 (37.9)	83 (42.6)	26 (13.3)
Difficulty sleeping	167	3	2 (1.2)	59 (35.3)	60 (35.9)	46 (27.5)
Dry mouth	90	2	11 (12.2)	50 (55.6)	20 (22.2)	9 (10)
Nausea	60	2	10 (16.7)	34 (56.7)	12 (20)	4 (6.7)
Vomiting	27	2	4 (14.8)	18 (66.7)	4 (14.8)	1 (3.7)
Change in the way food tastes	18	-	-	-	-	-
Lack of appetite	98	2	4 (4.1)	47 (48)	37 (37.8)	10 (10.2)
Weight loss	14	-	-	-	-	-
Difficulty swallowing	16	2	2 (12.5)	5 (31.3)	5 (31.3)	4 (25)
Cough	129	2	11 (8.5)	59 (45.7)	35 (27.1)	24 (18.6)
Shortness of breath	156	3	7 (4.5)	55 (35.3)	73 (46.8)	21 (13.5)
Swelling of arms/legs	82	-	-	-	-	-
Sweats	12	2	3 (25)	7 (58.3)	2 (16.7)	0.00
Itching	86	2	5 (5.7)	41 (47.1)	24 (27.6)	17 (19.5)
Mouth sores	8	-	-	-	-	-
Hair loss	44	-	-	-	-	-
Changes in skin	38	-	-	-	-	-
Feeling bloated	113	3	6 (5.3)	47 (41.6)	50 (44.2)	10 (8.8)
Problems with urination	79	2	1 (1.3)	40 (50.6)	27 (34.2)	11 (13.9)
Diarrhoea	14	2	2 (14.3)	10 (71.4)	2 (14.3)	0.00
Constipation	120	-	-	-	-	-
Problems with sexual interest or activity	23	4	0.00	7 (30.4)	6 (26.1)	10 (43.5)

Based on the MSAS developers, frequency is not assessed for eight symptoms – “I don’t look like myself”, “change in the way food tastes”, “weight loss”, “swelling of arms/legs”, “mouth sores”, “hair loss”, “changes in skin” and “constipation”

### Symptom severity

The severity of reported symptoms in MSAS was assessed asking participants to rate their severity of symptoms as slight, moderate, severe, and very severe. Table 4 presents pooled symptom severity. The proportion of symptoms rated as very severe ranged from 1% for dizziness to 30.4% for problems with sexual interest or activity. The most severe symptoms that were rated severe included shortness of breath (53.2%, n = 83), problems with urination (51.9%, n = 41), constipation (50.8%, n = 61), difficulty sleeping (49.7%, n = 83) and pain (46.2%, n = 90). In addition, 18 of the reported symptoms were rated as moderate severity including sweats (58.3%, n = 7), lack of appetite (52%, n = 51), vomiting (51.9%, n = 14), nausea (51.7%, n = 31), dry mouth (50%, n = 45) and difficulty swallowing (50%, n = 8). The symptom “I don’t look like myself” was the most frequent symptom that was rated as slight and was reported by 39.7% (n = 29).

**Table 4:** Symptom severity based on the Memorial Symptoms Assessment Scale

Symptom	N	M (*SD*)	Min-Max	Slight	Moderate	Severe	Very Severe
				n (%)			
Difficulty concentrating	75	1.9 (0.8)	1.0–4.0	25 (33.3)	37 (49.3)	11 (14.7)	2 (2.7)
Feeling nervous	97	2.0 (0.8)	1.0–4.0	17 (27.8)	45 (46.4)	23 (23.7)	2 (2.1)
Feeling sad	86	2.3 (0.9)	1.0–4.0	19 (22.1)	30 (34.9)	33 (38.4)	4 (4.7)
Worrying	103	2.1 (0.8)	1.0–4.0	29 (28.2)	42 (40.8)	29 (28.2)	3 (2.9)
“I don’t look like myself”	73	2.0 (1.0)	1.0–4.0	29 (39.7)	19 (26)	19 (26)	6 (8.2)
Lack of energy	207	2.5 (0.8)	1.0–4.0	17 (8.2)	83 (40.1)	90 (43.5)	17 (8.2)
Numbness/tingling in hands/feet	181	2.5 (0.8)	1.0–4.0	20 (11)	73 (40.3)	73 (40.3)	15 (8.3)
Dizziness	99	1.7 (0.7)	1.0–4.0	42 (42.4)	43 (43.4)	13 (13.1)	1 (1)
Pain	195	2.6 (0.8)	1.0–4.0	16 (8.2)	68 (34.9)	90 (46.2)	21 (10.8)
Difficulty sleeping	167	2.9 (0.8)	1.0–4.0	5 (3.0)	43 (25.7)	83 (49.7)	36 (21.6)
Dry mouth	90	2.1 (0.8)	1.0–4.0	20 (22.2)	45 (50)	21 (23.3)	4 (4.4)
Nausea	60	2.1 (0.8)	1.0–4.0	13 (21.7)	31 (51.7)	13 (21.7)	3 (5)
Vomiting	27	2.1 (0.8)	1.0–4.0	6 (22.2)	14 (51.9)	6 (22.2)	1 (3.7)
Change in the way food tastes	18	2.2 (1.2)	1.0–4.0	7 (38.9)	3 (16.7)	5 (27.8)	3 (16.7)
Lack of appetite	98	2.4 (0.7)	1.0–4.0	6 (6.1)	51 (52)	35 (35.7)	6 (6.1)
Weight loss	14	2.1 (1.0)	1.0–4.0	4 (28.6)	5 (35.7)	4 (28.6)	1 (7.1)
Difficulty swallowing	16	2.4 (1.0)	1.0–4.0	2 (12.5)	8 (50)	3 (18.8)	3 (18.8)
Cough	129	2.5 (0.9)	1.0–4.0	20 (15.5)	44 (34.1)	44 (34.1)	21 (16.3)
Shortness of breath	156	2.9 (0.7)	1.0–4.0	5 (3.2)	41 (26.3)	83 (53.2)	27 (17.3)
Swelling of arms/legs	82	2.3 (0.8)	1.0–4.0	12 (14.6)	39 (47.6)	27 (32.9)	4 (4.9)
Sweats	12	2.0 (0.9)	1.0–4.0	3 (25.0)	7 (58.3)	1 (8.3)	1 (8.3)
Itching	86	2.4 (0.8)	1.0–4.0	11 (12.8)	37 (43)	30 (34.9)	8 (9.3)
Mouth sores	8	2.6 (1.1)	1.0–4.0	1 (12.5)	3 (37.5)	2 (25)	2 (25)
Hair loss	44	2.5 (0.8)	1.0–4.0	4 (9.1)	19 (43.20)	17 (38.6)	4 (9.1)
Changes in skin	38	2.5 (0.8)	1.0–4.0	4 (10.5)	14 (36.8)	17 (44.7)	3 (7.9)
Feeling bloated	113	2.4 (0.8)	1.0–4.0	12 (10.6)	48 (42.5)	46 (40.7)	7 (6.2)
Problems with urination	79	2.6 (0.7)	1.0–4.0	6 (7.6)	27 (34.2)	41 (51.9)	5 (6.3)
Diarrhoea	14	2.2 (0.8)	1.0–3.0	3 (21.4)	5 (35.7)	6 (42.9)	0 (0.0)
Constipation	120	2.6 (0.7)	1.0–4.0	7 (5.8)	43 (35.8)	61 (50.8)	9 (7.5)
Problems with sexual interest or activity	23	3.0 (0.8)	2.0–4.0	0.00	6 (26.1)	10 (43.5)	7 (30.4)

### Symptom distress

The distress associated with symptoms was assessed. The participants rated the distress caused by their symptoms as “not at all”, “a little bit”, “somewhat”, “quite a bit” or “very much”. Distress differed along with the reported symptoms. Higher distress was mostly reported with the symptom “problems with sexual interest”. Participants reported very much distress with this symptom by 39.1% with a mean of 3 (*SD =* 1.1). Table 5 illustrates symptom distress as reported by the participants. Symptoms reported as quite a bit included shortness of breath (49.4%, n = 77), difficulty sleeping (47.3%, n = 79), pain (40.5%, n = 79), constipation (44.2%, n = 53) and changes in skin (36.8%, n = 14). Moreover, out of the 30 symptoms, 21 symptoms were reported as somewhat including lack of appetite (54.1%, n = 53), difficulty swallowing (50%, n = 8), itching (50%, n = 43) and lack of energy (49.8%, n = 103). Furthermore, few symptoms were frequently associated with little distress. These symptoms were weight loss (35.7%, n = 5), followed by “I don’t look like myself” (35.6%, n = 26), sweats (33.3%, n = 4) and change in the way food tastes (27.7%, n = 5).

**Table 5:** Symptom distress based on the Memorial Symptoms Assessment Scale

Symptoms	N	Mode	Not at all	A little bit	Somewhat	Quite a bit	Very much
			n (%)				
Difficulty concentrating	75	2	3 (4.0)	24 (32.0)	30 (40.0)	16 (21.3)	2 (2.7)
Feeling nervous	97	2	4 (4.1)	29 (29.9)	44 (45.4)	17 (17.5)	3 (3.1)
Feeling sad	86	2	2 (2.3)	21 (24.4)	32 (37.2)	25 (29.1)	6 (7.0)
Worrying	103	2	2 (1.9)	30 (29.1)	47 (45.6)	19 (18.4)	5 (4.9)
“I don’t look like myself”	73	1	8 (11.0)	26 (35.6)	22 (30.1)	12 (16.4)	5 (6.8)
Lack of energy	207	2	6 (2.9)	15 (7.2)	103 (49.8)	69 (33.3)	14 (6.8)
Numbness/tingling in hands/feet	181	2	4 (2.2)	19 (10.5)	85 (47.0)	60 (33.1)	13 (7.2)
Dizziness	99	2	16 (16.2)	35 (35.4)	37 (37.4)	10 (10.1)	1 (1.0)
Pain	195	3	6 (3.1)	16 (8.2)	72 (36.9)	79 (40.5)	22 (11.3)
Difficulty sleeping	167	3	3 (1.8)	9 (5.4)	42 (25.1)	79 (47.3)	34 (20.4)
Dry mouth	90	2	7 (7.8)	20 (22.2)	44 (48.9)	17 (18.9)	2 (2.2)
Nausea	60	2	3 (5.0)	17 (28.3)	26 (43.3)	11 (18.3)	3 (5.0)
Vomiting	27	2	0 (0.0)	7 (25.9)	12 (44.4)	7 (25.9)	1 (3.7)
Change in the way food tastes	18	1	3 (16.7)	5 (27.8)	3 (16.7)	4 (22.2)	3 (16.7)
Lack of appetite	98	2	2 (2.0)	8 (8.2)	53 (54.1)	29 (29.6)	6 (6.1)
Weight loss	14	1	3 (21.4)	5 (35.7)	1 (7.1)	4 (28.6)	1 (7.1)
Difficulty swallowing	16	2	1 (6.3)	1 (6.3)	8 (50.0)	3 (18.8)	3 (18.8)
Cough	129	2	10 (7.8)	11 (8.5)	48 (37.2)	37 (28.7)	23 (17.8)
Shortness of breath	156	3	2 (1.3)	6 (3.8)	42 (26.9)	77 (49.4)	29 (18.6)
Swelling of arms/legs	82	2	0 (0.0)	13 (15.9)	34 (41.5)	30 (36.6)	5 (6.1)
Sweats	12	1	2 (16.7)	4 (33.3)	4 (33.3)	1 (8.3)	1 (8.3)
Itching	86	2	4 (4.7)	9 (10.5)	43 (50.3)	23 (26.7)	7 (8.1)
Mouth sores	8	2	0.00	2 (25)	3 (37.5)	1 (12.5)	2 (25.0)
Hair loss	44	2	5 (11.4)	6 (13.6)	17 (38.6)	13 (29.5)	3 (6.8)
Changes in skin	38	3	1 (2.6)	8 (21.1)	11 (28.9)	14 (36.8)	4 (10.5)
Feeling bloated	113	2	2 (1.8)	14 (12.4)	55 (48.7)	37 (32.7)	5 (4.4)
Problems with urination	79	2	1 (1.3)	8 (10.1)	33 (41.8)	32 (40.5)	5 (6.3)
Diarrhoea	14	2	0 (0.0)	4 (28.6)	6 (42.9)	4 (28.6)	0 (0.0)
Constipation	120	3	1 (0.8)	9 (7.5)	48 (40.0)	53 (44.2)	9 (7.5)
Problems with sexual interest or activity	23	4	1 (4.3)	0 (0.0)	7 (30.4)	6 (26.1)	9 (39.1)

### Symptom treatment

Treatment and its effectiveness are critical dimensions of symptom experience. Table 6 shows symptom treatment and effectiveness. The results indicated that four symptoms were not treated at all including feeling sad, “I don’t look like myself”, dry mouth and a change in the way food tastes. Furthermore, in 11 symptoms, 10% or fewer participants reported receiving treatment. The symptoms that were reported to have the highest treatment rate included shortness of breath (66.7%, n = 104), pain (61.3%, n = 119), cough (52.7%, n = 68), mouth sores (50%, n = 4) and itching (46.5%, n = 40). Although symptoms that were reported to have the lowest treatment, the rates included feeling nervous (1%, n = 1), worrying (1%, n = 1), lack of energy (1%, n = 2), difficulty concentrating (1.3%, n = 1) and hair loss (2.3%, n = 1). Treatment effectiveness for these symptoms varied from successful, slightly successful and failure. Further, successful treatment was reported as 61.5%, n = 64 for shortness of breath treatment, 60.5%, n = 72 for pain treatment, 50%, n = 20, 50%, n = 2 for both itching and mouth sores treatment and (43.3%, n = 29) for cough treatment. Treatment effectiveness was rated as slightly successful for the treatment of several symptoms including feeling nervous (100%, n = 1), worrying (100%, n = 1), lack of energy (100%, n = 2), vomiting (100%, n = 1) and weight loss (100%, n = 1). While treatment was reported as failed for seven symptoms including problems with sexual interest or activity (100%, n = 1), mouth sores (50%, n = 2), itching (25%, n = 10), changes in skin (23.1%, n = 3) and constipation (17.4%, n = 8).

**Table 6:** Symptom treatment and effectiveness of treatment as perceived by participants

Symptom	N	Treatment		Treatment effectiveness		
		Yes	No	Successful	Slightly successful	Failed
		n (%)		n (%)		
Difficulty concentrating	75	1 (1.3)	74 (98.7)	1 (100.0)	0 (0.0)	0 (0.0)
Feeling nervous	97	1 (1.0)	96 (99.0)	0 (0.0)	1 (100.0)	0 (0.0)
Feeling sad	86	0 (0.0)	86 (100.0)	-	-	-
Worrying	103	1 (1.0)	102 (99.0)	0 (0.0)	1 (100.0)	0 (0.0)
“I don’t look like myself”	73	0 (0.0)	73 (100.0)	-	-	-
Lack of energy	207	2 (1.0)	204 (99.0)	0 (0.0)	2 (100.0)	0 (0.0)
Numbness/tingling in hands/feet	181	17 (9.4)	164 (90.6)	6 (35.3)	9 (52.9)	2 (11.8)
Dizziness	99	7 (7.1)	92 (92.9)	6 (85.7)	1 (14.3)	0 (0.0)
Pain	195	119 (61.3)	75 (38.7)	72 (60.5)	37 (31.1)	10 (8.4)
Difficulty sleeping	167	30 (18.0)	137 (82.0)	21 (70.0)	6 (20.0)	3 (10)
Dry mouth	90	0 (0.0)	90 (100.0)	-	-	-
Nausea	60	7 (11.7)	53 (88.3)	2 (28.6)	5 (71.4)	0 (0.0)
Vomiting	27	1 (3.7)	26 (96.3)	0 (0.0)	1 (100.0)	0 (0.0)
Change in the way food tastes	18	0.00	18 (100.0)	-	-	-
Lack of appetite	98	3 (3.1)	95 (96.9)	3 (100.0)	0 (0.0)	0 (0.0)
Weight loss	14	1 (7.1)	13 (92.9)		1 (100.0)	
Difficulty swallowing	16	2 (12.5)	14 (87.5)	2 (100.0)	0 (0.0)	0 (0.0)
Cough	129	68 (52.7)	61 (47.3)	29 (43.3)	32 (47.8)	6 (9.0)
Shortness of breath	156	104 (66.7)	52 (33.3)	64 (61.5)	39 (37.5)	1 (1.0)
Swelling of arms/legs	82	31 (37.8)	51 (62.2)	19 (61.3)	12 (38.7)	0 (0.0)
Sweats	12	2 (16.7)	10 (83.3)	1 (50.0)	1 (50.0)	0 (0.0)
Itching	86	40 (46.5)	46 (53.5)	20 (50.0)	10 (25.0)	10 (25.0)
Mouth sores	8	4 (50.0)	4 (50.0)	2 (50.0)	0 (0.0)	2 (50.0)
Hair loss	44	1 (2.3)	43 (97.7)	1 (100.0)	0 (0.0)	0 (0.0)
Changes in skin	38	13 (34.2)	25 (65.8)	7 (53.8)	3 (23.1)	3 (23.1)
Feeling bloated	113	25 (22.1)	88 (77.9)	10 (40.0)	11 (44.0)	4 (16.0)
Problems with urination	79	26 (32.9)	53 (67.1)	14 (53.8)	12 (46.2)	0 (0.0)
Diarrhoea	14	3 (21.4)	11 (78.6)	1 (33.3)	2 (66.7)	0 (0.0)
Constipation	120	46 (38.3)	74 (61.7)	15 (32.6)	23 (50)	8 (17.4)
Problems with sexual interest or activity	23	1 (4.30)	22 (95.7)	0 (0.0)	0 (0.0)	1 (100.0)

## Discussion

### Symptom prevalence

The results of this study indicate that symptoms were prevalent among patients with chronic obstructive pulmonary diseases (COPD), chronic heart failure (CHF) and end-stage renal disease (ESRD). However, each disease had a different set of highly prevalent symptoms. When comparing the study results with previous studies, it is important to point out that there are very limited up-to-date studies that have assessed symptoms and reported pooled prevalence for more than one chronic disease. Therefore, the results of the study are compared to previous studies that included at least one of the selected chronic diseases.

First, the most common symptoms among participants with COPD were shortness of breath, lack of energy, pain, cough, and difficulty sleeping. These results are partially consistent with what has been reported in previous studies regarding symptom prevalence among patients with COPD [9, 21, 22]. For example, Melhem et al. [9] found that the most prevalent symptoms among COPD patients were problems with sexual activity and interest, cough, shortness of breath, lack of appetite,, and lack of energy. In another study, Miravitlles et al. [22] found that the most prevalent symptoms among patients with COPD were breathlessness, coughing, coughing up mucus, wheezing, and chest tightness. Although the results of this study differ from previous studies in terms of the highly prevalent symptoms, the results were in agreement with those reporting shortness of breath, coughing, and lack of energy as the top symptoms reported among COPD patients.

Second, the most common symptoms among participants with ESRD were pain, lack of energy, numbness, itching, and difficulty sleeping. This was slightly different from what has been previously reported [10, 12, 23, 24]. For example, in a study conducted in Saudi Arabia, Almutary et al. [10] found that the most prevalent symptoms among patients with ESRD were fatigue, bone pain, itching, and decreased appetite. In addition, Bonner et al., (2018) found that the most prevalent symptoms among patients with ESRD were fatigue, dry mouth and skin, and bone, or joint pain. Moreover, Chaiviboontham et al. [23] found that the most prevalent symptoms among ESRD patients were itching, skin dryness, muscle pain, dry mouth, and muscle cramps. Based on the above, most of the studies mutually reported pain (any form of pain) and fatigue as the most prevalent symptoms.

Third, the study revealed that the most prevalent symptoms among participants with CHF were, lack of energy, numbness, pain, difficulty sleeping and swelling of arms/legs. This result differs slightly from those reported in previous studies [25, 26]. However, some mutual symptoms were reported, and the difference was in the ranking. For example, in a study conducted by Haedtke et al., [25], the most prevalent symptoms among patients with CHF were non-cardiac pain, shortness of breath, lack of energy, feeling drowsy, and numbness and tingling. In another study, Lokker et al. [26] assessed symptoms among patients with advanced heart failure and found that the most prevalent symptoms were shortness of breath, worry, feeling irritable, feeling drowsy, and feeling sad. The lack of energy symptom was present in the highly prevalent symptoms in the current study, as well as in previous studies.

In the current study, the set of highly prevalent symptoms among patients with chronic chronic obstructive pulmonary diseases (COPD), chronic heart failure (CHF), and end-stage renal disease (ESRD) was partially consistent with what has been reported in previous studies for several reasons. First, the assessment tool used in the current study was the MSAS, which is a multidimensional symptom assessment tool, while some studies used disease-specific tools, such as the night-time, morning, and daytime symptoms of the COPD questionnaire, which was used by Miravitlles et al. [22]. These tools differ in the number of assessed symptoms, time of assessment, and the symptom nomenclature. Second, chronic diseases progress over the time from mild to severe and symptom prevalence is greatly affected by the disease severity as it increases with the increased severity and in the current study, disease severity was not considered. Third, the sample size of the study affects the precision of the results, the larger the sample size, the better the precision of the results. Although the current study had an adequate number of participants for the total study, the number of participants with COPD (n = 100) was relatively small. Future studies should consider the stage and severity of the disease and enrol a larger number of participants.

Overall, it is obvious that patients with selected chronic diseases suffer from several and varying sets of symptoms, so it is necessary to frequently evaluate patients’ symptoms and follow up on these symptoms while patients are at home. This assessment needs to be tailored and individualised for each patient, since each disease can result in many different symptoms that can even vary between patients with the same diseases.

### Symptom severity

Regarding symptom severity, the results indicate that the most severe symptoms reported by patients with chronic obstructive pulmonary diseases (COPD), chronic heart failure (CHF) and end-stage renal disease (ESRD) were shortness of breath, problems with urination, constipation, difficulty sleeping and pain. These results were partially consistent with the set of the most severe symptoms reported by Melhem et al., [9]; Almutary et al., [10]; Bonner et al., [12]; Chaiviboontham et al., [23]; and Haedtke et al., [25]. For example, Melhem et al. [9] stated that the most severe symptoms among patients with COPD were difficulty swallowing, difficulty concentrating, difficulty sleeping, problems with sexual interest and activity, and lack of appetite. Like previous studies [9, 10, 12, 23, 25], the results of this study indicate that difficulty sleeping, pain, shortness of breath and constipation were the symptoms ranked as severe by the patients.

This highlights the complexity of symptoms experience; it is multidimensional and seems to be difficult to achieve, however, implementing appropriate symptom management guidelines in clinical practise is highly required to reduce symptoms severity. The importance of re-evaluation and follow-up on the presence and severity of symptoms with the patients needs to be stressed.

### Symptom frequency

The current study revealed that among the reported symptoms, the most frequent (constantly) symptom was problems with sexual interest or activity. Of those who had the symptom, 43.5% reported it as constantly occurring. When comparing these results with those of older studies, it must be pointed out that symptom frequency was not explored in most of the previously published studies. However, the current study results were like the results of the few studies that examined symptom frequency [9, 10, 21, 25]. For example, Melhem et al., [9] found that the highly frequent symptoms among COPD patients included problems with sexual interest or activity, cough, shortness of breath, lack of appetite and lack of energy. In addition, Eckerblad et al., [21] found that the most frequent and constant symptoms among patients with stable COPD were shortness of breath, dry mouth, difficulty sleeping, lack of energy and feeling drowsy.

### Symptom distress

This study indicated that a higher level of distress was reported primarily with symptoms of sexual interest or activity. The participants reported very much distress with this symptom by (39.1%) with a mean of 2.96 (*SD =* 1.07). In addition, symptoms frequently reported as quite a bit of distress included shortness of breath, difficulty sleeping, pain, constipation, and changes in skin. These symptoms were found to be associated with higher distress compared to other symptoms. Although the symptom problems with sexual interest was not prevalent or severe, it was highly distressing as reported by the patients. Reflecting on this, the sample in this study could be sexually active and these diseases affect the physical aspect of the patients’ life. Hence, it might explain the high distress associated with lacking sexual ability. Further, symptom severity usually treated as the most important symptom dimension and equivalent to other symptom dimensions. For example, if a symptom is severe, it must also be distressing. But these results show that the symptom dimensions are not equivalent, symptom might be not prevalent, but it could be highly distressing. Therefore, this requires a comprehensive assessment of symptoms considering all dimensions to achieve optimal treatment.

This result deserves further exploration using a qualitative research approach. These results were consistent with what has been found in previous studies [10, 21, 25]. For example, Eckerblad et al. [21] found that the most distressing symptoms among patients with stable COPD were shortness of breath, lack of energy, pain, and difficulty sleeping. Furthermore, Haedtke et al. [25] reported that the most distressing symptoms among patients with advanced CHF included lack of energy, pain (other than chest pain) and shortness of breath.

### Symptom management as perceived by patients

The current study assessed symptom management, specifically treatment and treatment effectiveness as perceived by patients. The results indicate that most of the symptoms were not adequately treated as reported by the patients, and some of them even received no treatment. It was obvious that physical symptoms, such as shortness of breath, pain, cough, mouth sores, and itching, received most of the attention and are most likely to be treated compared to psychological symptoms, such as difficulty concentrating, feeling nervous, feeling sad, worrying, and “I don’t look like myself”. These findings are consistent with older studies [27–29]. For example, Laville et al. [28] found that patients with ESRD frequently received inappropriate drug prescriptions. In another study conducted in Oman by Hanbali et al. [27], the use of guideline-based cardiovascular medications in heart failure patients was found to be low in Oman. Additionally, the dose was highly inadequate for patients with heart failure [27]. However, these studies did not differentiate between physical and psychological symptom treatment adequacy. Inappropriate symptom management can have several consequences for patients and healthcare systems. For example, according to Rothrock et al., [5], symptoms can increase patients’ visits to healthcare settings, which may increase the costs. In addition, it can cause patient suffering and loss of hope. Furthermore, the burden of symptoms can decrease the productivity of patients and reduce the level of their quality of life [13]. Finally, psychological symptoms were found to receive less attention in terms of assessment and management; healthcare providers should consider assessing and managing psychological symptoms as a priority rather than focussing only on physical symptoms. However, symptom management is a main component of palliative care that was expanded in scope to include patients with incurable non-malignant disease such as CHF, COPD, and ESRD. Thus, introducing such services for those patients may enhance symptoms management, patient outcomes and reduce patients suffering.

### Limitations

The findings of the study need to be interpreted considering the following limitations. One of the important determinants of symptoms prevalence, severity and distress is the stage and severity of the selected chronic diseases, which were not taken into consideration in the current study. This may affect the study results and the dimension of the dilute symptoms as the patients were recruited at different stages. To enhance the external validity and generalisability of the findings, future studies should recruit participants from different stages and with varied disease severity. In addition, the number of participants recruited from each disease group was relatively small despite the adequacy of the overall sample size. Increasing the number of participants in each disease group will help better understand the symptom profile and improve the external validity of the results. Also, we included only patients with ESRD CHF and COPD and this may introduce selection bias. Furthermore, the MSAS assessment tool assesses symptoms by requesting patients to recall symptoms over the past week; therefore, they may not recall them properly, which leads to recall bias. Future studies may consider assessing the current symptom experience.

## Conclusion

The findings of the current study showed that symptoms were prevalent and that some symptoms were frequent, severe, and highly distressing. In addition, treatment as perceived by patients was inadequate. Obviously, psychological symptoms received less attention in treatment compared to physical symptoms, which can negatively affect patients’ health. Understanding the experience of symptoms of patients with chronic diseases is essential when planning and implementing management plans. One of the mainstays for the management of symptoms can be the introduction of palliative care. Providing palliative care to these patients can alleviate their suffering and improve their quality of life. In addition, designing chronic disease self-management programmes can make a difference to patients’ life.

## Data Availability

The data sets analysed during the current study are available from the first author [Fatima ALHosni] on reasonable request.

## Abbreviations

CDC: Centre of Disease Control and Prevention
US: United States
COPD: Chronic Obstructive Pulmonary Diseases
CHF: Chronic Heart Failure
ESRD: End-Stage Renal Disease
DDS: Demographic Data Sheet
MSAS: Memorial Symptoms Assessment Scale
